# Too Few, Too Weak: Conflict of Interest Policies at Canadian Medical Schools

**DOI:** 10.1371/journal.pone.0068633

**Published:** 2013-07-04

**Authors:** Adrienne Shnier, Joel Lexchin, Barbara Mintzes, Annemarie Jutel, Kelly Holloway

**Affiliations:** 1 Health Policy and Equity, School of Health Policy and Management, York University, Toronto, Canada; 2 School of Health Policy and Management, York University, Toronto, Canada; 3 University Health Network, Toronto, Canada; 4 Department of Family and Community Medicine, Faculty of Medicine, University of Toronto, Toronto, Canada; 5 School of Population and Public Health, University of British Columbia, Vancouver, Canada; 6 Graduate School of Nursing Midwifery and Health, Victoria University of Wellington, Wellington, New Zealand; 7 Department of Sociology, York University, Toronto, Canada; State University of New York, Oswego, United States of America

## Abstract

**Introduction:**

The education of medical students should be based on the best clinical information available, rather than on commercial interests. Previous research looking at university-wide conflict of interest (COI) policies used in Canadian medical schools has shown very poor regulation. An analysis of COI policies was undertaken to document the current policy environment in all 17 Canadian medical schools.

**Methods:**

A web search was used to initially locate COI policies supplemented by additional information from the deans of each medical school. Strength of policies was rated on a scale of 0 to 2 in 12 categories and also on the presence of enforcement measures. For each school, we report scores for all 12 categories, enforcement measures, and summative scores.

**Results:**

COI policies received summative scores that ranged from 0 to 19, with 0 the lowest possible score obtainable and 24 the maximum. The highest mean scores per category were for disclosure and ghostwriting (0.9) and for gifts and scholarships (0.8).

**Discussion:**

This study provides the first comprehensive evaluation of all 17 Canadian medical school-specific COI policies. Our results suggest that the COI policy environment at Canadian medical schools is generally permissive. Policy development is a dynamic process. We therefore encourage all Canadian medical schools to develop restrictive COI policies to ensure that their medical students are educated based on the best clinical evidence available, free of industry biases and COI relationships that may influence the future medical thinking and prescribing practices of medical students in Canada once they graduate.

## Introduction

Conflicts of interest with industry may occur in medical education in the classroom, in the conduct and reporting of research, at the bedside, and in the treatment of patients. The education of medical students should be based on the best clinical information available, unbiased by the commercial interests of industries marketing pharmaceutical or other health products. In many Canadian medical schools, students are taught by faculty who work in partnership with industry, e.g., receive research grants from companies, serve on companies’ speakers’ bureaus or advisory committees, or own shares in companies. [1] The financial relationships of faculty with industry may affect, or reasonably appear to affect, the integrity of their academic or publishing interests, professional medical opinions, and the information that they disseminate to medical students. [2], [3] These relationships between medical faculty and industry represent conflicts of interest (COI) and compromise not only the public’s confidence and trust in medical researchers and universities, [2], [4], [5] but also the potential for robust, evidence-based clinical education for medical students. [6].

When medical school faculty members have ties with, or financial interest in, pharmaceutical companies, they are more likely to report results that are favourable to the sponsoring companies. [2] Faculty with financial COI tend to publish significantly more, and at a higher rate, than faculty without industry relationships. [7] At the same time, these faculty members are also more likely to conduct lower quality, but more commercializable research, as compared with those who undertake independently funded research. [2], [8] Quality of research is evaluated based on the following criteria: whether clinical trial data is selectively reported, the medication being tested in a trial is compared to one that is known to be inferior, inappropriate doses of a competitor drug are used in a trial, and the length of clinical trials is altered to produce data that is favorable to the sponsors’ drugs, among other methods. [2], [8].

COI relationships are present not only in the classroom, but also surface when industry provides resources to medical schools. Although corporate pharmaceutical funding for education may offer educational opportunities for students, these programs tend to provide students with industry-friendly information, which can compromise clinical judgment if it is at odds with the scientific evidence. For example, between 2002 and 2006, the pain management course for medical and other health science professional students held at University of Toronto was partly funded by grants from Purdue Pharma LP, the maker of OxyContin. As part of the course, a chronic pain management book that was funded and copyrighted by Purdue Pharma was distributed to the students by a lecturer who was external to University of Toronto and had financial ties to Purdue Pharma. Concerns were raised that some of the contents of the book were not consistent with the current best evidence for narcotic medication administration. [9] Without effective, stringent COI policies at medical schools to regulate such interactions between faculty, students, and industry, medical students are subject to direct or indirect interactions with industry, as well as industry resources, that have the potential to influence their future medical thinking and prescribing practices.

The implementation of COI policies has been effective in altering the future prescribing practices of medical residents. Epstein and colleagues conducted an analysis of the antidepressant prescribing practices of 1652 graduates from 162 psychiatric residency programs in the US before 2001 and after these programs adopted COI policies in 2008. [10] The authors found that residents who graduated before the introduction of COI policies in 2001 tended to prescribe less appropriately than 2008 graduates, where inappropriate prescribing was defined as prescribing heavily marketed and brand reformulated antidepressants (e.g., extended release products) at a higher rate. Furthermore, 2008 residents who graduated from programs with maximally restrictive COI policies prescribed these drugs significantly less often than 2008 graduates from programs with minimally restrictive COI policies.

The Association of Faculties of Medicine of Canada has voted to support the 2008 report by the Association of American Medical Colleges [6], [11] to better manage and, when necessary, prohibit interactions between academics and industry that can create COI and undermine professionalism standards. Previous research on COI policies as applied to Canadian medical schools has shown very poor regulation. Mathieu and colleagues used the American Medical Students Association (AMSA) scorecard to analyze COI policies at Canadian universities that host medical schools. [12] They found that the university-wide policies were generally weak in the areas of faculty-industry relationships, samples, sales representatives, on-site and off-site training, industrial relationships, and educating students about COI.

However, the scope of Mathieu and colleagues’ study was limited because the authors only analyzed university-wide COI policies and not those specific to medical schools. Further, they omitted the Northern Ontario School of Medicine (NOSM) from their analysis. In addition, they did not contact the universities directly and only relied on institutional policies found via a web search. Finally, only a single coder evaluated the COI policies. To address these limitations, we undertook an analysis of COI policies at both the university and faculty levels to document the current COI policy environment in all 17 Canadian medical schools.

## Methods

A list of all 17 medical schools (14 English language and 3 French language) in Canada was obtained from the web site of the Association of Faculties of Medicine of Canada (AFMC) <http://www.afmc.ca/faculties-e.php>. The web site of each of the schools was searched in late July 2011 for policies related to COI or documents interpreting policies using the terms “policy”, “policies”, “conflict-of-interest”, “conflicts-of-interest” and “COI” in English, and “politique” and “conflit d’intérêts” in French. The name of each policy and the latest of either the date of adoption or the date of the policy’s most recent review were recorded. After a preliminary list of policies for each school was assembled, an e-mail with the list of policies in English or French, as appropriate, was sent to each dean explaining the purpose of the study and requesting confirmation that this list contained the pertinent policies for the particular medical school. These emails also requested that the deans send us any additional policies we might have overlooked, or draft policies not yet in place. The deans were informed that we were only interested in publicly available policies and while respondents’ names would be confidential, the medical schools and their policies would be identified in any subsequent publication. Two reminder emails were sent at one-month intervals. We did not search for, request, or analyze policies from affiliated teaching hospitals.

Policies that were approved as of the end of September 2011 were analyzed. A grading system was modified from those that were already used by AMSA, [13] Chimonas and colleagues, [14] and Mason and Tattersall [15] for 12 different categories:

- gifts (including meals)
- consulting relationships (excluding scientific research and speaking)
- industry-funded speaking relationships and speakers’ bureaus
- honoraria, ghostwriting
- disclosure
- industry sales representatives
- on-site education activities
- compensation for travel or attendance at off-site lectures and meetings
- industry support for scholarships and funds for trainees
- medical school curriculum (or other documentation of educational objectives and course content)
- samples

AMSA uses a 0 to 3 scoring system where a score of 0 indicates that schools failed to respond to its request to send their policies. Since we initially identified policies using a web search, AMSA’s definition of what constituted a score of 0 was not relevant. Both AMSA and Mason and Tattersall regard a permissive policy as equivalent to the absence of a policy. We graded each category on a scale of 0 to 2, where 0 = no policy or permissive, 1 = moderate, and 2 = restrictive. (See Appendix S1 for the detailed scoring criteria for each individual category.) In addition, we scored enforcement measures: is it clear that a party is responsible for general oversight to ensure compliance and is it clear there are sanctions for noncompliance? Each of these enforcement measures was scored either “yes” or “no.” We did not attempt to identify if policies had been violated or to grade the severity of sanctions.

Scoring was done by two groups of two people, one for English (AS, KH) and one for French language schools (BM, AJ). Each person independently scored the policies and then compared results within their group. Disagreements were resolved through discussion. Once the scoring was completed, a follow-up e-mail in the appropriate language was sent to each dean. This email included the preliminary scoring for the medical school along with the policies that we used to obtain the score, an explanation of how each area was scored and a request that the dean review the scores for accuracy and notify us if he or she felt that a score was inaccurate. We also requested that the deans send us any new policies developed since the initial contact, but noted that the scores would be based on policies in place as of the end of September 2011. We asked the deans to respond within one month, and if we had not heard from them at that point, two further e-mail reminders were sent at one-month intervals.

After a response from the deans, the scores were reviewed by the original set of scorers, and a final set of scores was derived for each school. Similarly to Chimonas and colleagues, [14] we summed the scores in the first 12 individual categories for each school to come up with a summative score. Each category was weighted equally since each was identified as vital by a combination of the American Board of Internal Medicine-Institute on Medicine as a Profession (ABIM-IMAP), American Association of Medical Colleges (AAMC), and the Institute of Medicine (IOM). [14] We view this weighing system as also being applicable to the Canadian situation since the AFMC has endorsed the report by the AAMC on industry funding of medical education. [16] For the enforcement categories, the number of “yes” and “no” for each school was summed. We report scores for each category for each school, the summative scores for each school, and the mean for each category.

Since we collected only publicly available information about medical schools’ COI policies, the Human Participants Review Committee at York University, which approved this project, waived the requirement for informed consent from the deans of the schools that we contacted.

## Results

Of 17 medical schools contacted, 15 responded to the initial request for policies. Via web searches and responses from deans’ offices, we found a total of 50 policies and documents interpreting policies (collectively referred to as policies). Schools had as few as zero (NOSM) and as many as 8 relevant policies (University of British Columbia) per school. In addition, two deans sent us course outlines used in the teaching of COI to medical students. The dates of 16 policies were either not given (9) or were unclear (7). For the other 34 policies, seven were more than 10 years old (one dated back to 1976), while 12 were passed within two years of September 2011 (Table 1). Twenty-one policies were at the medical school level and the remainder (29) were university-wide.

**Table 1:** Policies per school and date of each policy.

School	Name of policy	Date of adoption/most recent review
Dalhousie University	Guidelines for the relationship between medical education and health related industries (S*)	September 2011
	Policy on conflict of interest (U†)	June 24, 2002
Laval Université	Normes de gestion des Fonds de soutien à l’enseignement des programmes de residence (S)	June 18, 2010
	Politique de la Faculté de médicine sur les relations entre les membres de la Faculté de médecine de l’Université Laval et les entreprises privées relativement aux activités et aux programmes de formation sous la responsabilité de la Faculté (S)	December 19, 2008
	Politique sur l’intégrité en recherché et création et sur les conflits d’intérêts (U)	May 20, 2009
McGill University	Code of conduct: faculty of medicine (S)	No date given
	Handbook: student rights and responsibilities (U)	2010
	Recognizing conflicts (U)	No date given
	Regulations concerning investigation of research misconduct (U)	May 25, 2010
	Regulation on conflict of interest (U)	June 15, 2009
McMaster University	Guidelines regarding management of commercial/private sector/government relationships in research and education (S)	January 23, 2008
	Joint intellectual property policy (U)	May 27, 1998
	Policy on support of continuing education events from commercial sources (S)	2007
	Postgraduate education guidelines for interaction with the pharmaceutical industry (S)	No date given
	Statement on consulting policy and procedures (U)	January 14, 1976
	Statement on conflict of interest in research (U)	March 11, 2009
Memorial University of Newfoundland	Conflict of interest (U)	March 31, 2011
	Integrity in scholarly research (U)	February 12, 2001
	Procedure for investigation reports of misconduct in research (U)	No date given
Northern Ontario Medical School	No policies	No policies
Queens University	Physicians and industry – conflicts of interest (S)	Date uncertain
	Policy of disclosure on conflict of interest (U)	August 17, 2001
Université de Montréal	Règlement sur les conflicts d’intérêts (U)	November 24, 2009
Université de Sherbrooke	Guide sur les relations entre les milieux de formation en santé et les entreprises (S)	March 17, 2010
	Politique, règles et proc édures sur l’intérité en recherché et sur les conflicts d’intérêts (U)	May 30, 2006
University of Alberta	Conflict of interest and conflict of commitment reporting and assessment policy (U)	November 16, 2009
	Conflict policy – conflict of interest and commitment and institutional conflict (U)	June 26, 2009
University of British Columbia	Conflict of interest/commitment declaration – steps (U)	Date uncertain
	Conflict of interest and conflict of commitment (S)	November 2007
	Dean’s COI/COC review committee (S)	November 7, 2006
	Definitions (U)	Date uncertain
	Duty to disclose (U)	Date uncertain
	Frequently asked questions (FAQs) (U)	Date uncertain
	Managing conflicts – what to do (U)	Date uncertain
	Reviewer resources (U)	Date uncertain
University of Calgary	Conflict of interest policy (U)	September 1, 1987
	Disclosure of potential financial conflict of interest for use by planning committees for continuing medical education and professional development programs (S)	No date given
	Disclosure of potential financial conflict of interest for use by speakers for continuing medical education and professional development programs (S)	No date given
	Research policy for integrity in scholarly activity (S)	December 9, 1992
University of Manitoba	Interactions between the University of Manitoba’s Faculty of Medicine and the pharmaceutical, biotech, medical device, and hospital and research equipment and supplies industries (“Industry”) (S)	June 3, 2009
	Policy on industry relations (S)	No date given
University of Ottawa	Conflict of interest – members of staff (U)	October 20, 2009
	Interacting with industries and outside agencies in a teaching environment (U)	November 19, 2008
	Interactions between the Faculty of Medicine and the pharmaceutical, biotechnology, medical device, and hospital and research equipment and supplies industries (S)	September 2011
	Standards of ethical and professional behaviour (S)	no date given
University of Saskatchewan	Conflict of interest (U)	December 12, 2008
	Research integrity policy (U)	June 17, 2010
University of Toronto	CEPD policy on support of University of Toronto sponsored continuing education activities from commercial sources (S)	November 15, 2004
	Policy on conflict of interest – academic staff (U)	June 22, 1994
Western University (formerly University of Western Ontario)	Policy and guidelines for interactions between Schulich School of Medicine and Dentistry and pharmaceutical, biotech, medical device and research equipment supplies industry (“Industry”) (S)	June 4, 2010
	Recommendations and frequently asked questions (FAQs) (S)	no date given

* S = School-specific policy.

† U = University-wide policy.

Eleven schools responded when we asked them to review their initial scores, resulting in the revision of scores for five schools that provided policies that we had initially overlooked or that they had not initially sent us. In addition, two schools informed us that they had put in place new policies since our initial survey, while seven were in the process of developing or updating policies.

COI policies received summative scores that ranged from 0 (NOSM) to 19 (Western University, formerly University of Western Ontario), where 0 was the lowest score possible and 24 was the maximum score (Table 2). Twelve of the 17 schools scored less than 12/24 (50% of the maximum) and only one scored more than 18/24 (75% of maximum). Cumulative scores of 5 or less reflected ratings of mostly 0 (no policy or permissive) for each category, whereas cumulative scores of 8 or more reflected ratings of 1 or 2 (moderate or restrictive, respectfully) for most categories. The highest mean scores were assigned to disclosure and ghostwriting (0.9) and for gifts and scholarships (0.8). Policies on sampling received the lowest average score (0.2), followed by policies on sales representatives (0.3) and speaking and curriculum (0.4). No school had a restrictive policy that applied to samples. Of note, no category received a mean score of 1 or better (Table 3). Many COI policies with a rating less than 2 for disclosure failed to require disclosure of both past and present financial ties with industry on a publicly-available website and/or disclosure of any relationships to patients when this relationship may represent a COI.

**Table 2:** Medical schools and scoring for individual category.

School	Strength of policy			Total score (percent of maximum)	Enforcement	
					Party responsible for enforcement	Sanctions for violations
	No policy or permissive	Moderate policy	Restrictive policy			
	(score = 0)	(score = 1)	(score = 2)		(Yes/No)	(Yes/No)
Western University (formerly University of Western Ontario)	honoraria	curriculum, sales representatives, samples	compensation, consulting, disclosure, ghostwriting, gifts, on-site education, scholarships, speaking	19 (79)	Yes	Yes
University of Manitoba	curriculum, samples	compensation, disclosure, honoraria, sales representatives	consulting, ghostwriting, gifts, on-site education, scholarships, speaking	16 (67)	Yes	Yes
University of Ottawa	consulting	compensation, curriculum, disclosure, on-site education, samples, sales representatives, speaking	ghostwriting, gifts, honoraria, scholarships	15 (63)	Yes	Yes
Dalhousie University	samples, speaking	compensation, consulting, curriculum, disclosure, on-site education, sales representatives	ghostwriting, gifts, honoraria, scholarships	14 (58)	Yes	Yes
Université de Sherbrooke	curriculum, speaking	consulting, disclosure, gifts, honoraria, on-site education, sales representatives, samples	compensation, ghostwriting, scholarships	13 (54)	Yes	Yes
Laval Université	ghostwriting, sales representatives, samples, speaking	consulting, disclosure, gifts, honoraria, on-site education	compensation, curriculum, scholarship	11 (46)	Yes	Yes
University of Toronto	curriculum, ghostwriting, gifts, sales representatives, samples	compensation, disclosure, honoraria, on-site education, sales representatives, samples	consulting	8 (33)	Yes	No
McMaster University	compensation, curriculum, ghostwriting, gifts, sales representatives, samples, scholarships, speaking	consulting, disclosure, honoraria	on-site education	5 (21)	Yes	No
University of British Columbia	compensation, curriculum, ghostwriting, on-site education, sales representatives, samples, scholarships	consulting, disclosure, gifts, honoraria, speaking		5 (21)	Yes	No
McGill University	compensation, consulting, gifts, honoraria, on-site education, sales representatives, samples, scholarships, speaking	curriculum, disclosure	ghostwriting	4 (17)	Yes	Yes
Memorial University of Newfoundland	compensation, curriculum, ghostwriting, honoraria, on-site education, sales representatives, samples, scholarships, speaking	consulting, disclosure, gifts		3 (13)	Yes	Yes
University of Calgary	compensation, consulting, curriculum, gifts, honoraria, on-site education, sales representatives, samples, scholarships, speaking	disclosure	ghostwriting	3 (13)	Yes	No
University of Saskatchewan	compensation, consulting, curriculum, gifts, honoraria, on-site education, sales representatives, samples, scholarships, speaking	disclosure	ghostwriting	3 (13)	Yes	Yes
Université de Montréal	compensation, consulting, curriculum, ghostwriting, honoraria, on-site education, sales representatives, samples, scholarships, speaking	disclosure, gifts		2 (8)	Yes	No
Queens University	compensation, consulting, curriculum, disclosure, ghostwriting, gifts, honoraria, on-site education, sales representatives, samples, speaking	scholarships		1 (4)	No	No
University of Alberta	compensation, consulting, curriculum, ghostwriting, gifts, honoraria, on-site education, sales representatives, samples, scholarships, speaking	disclosure		1 (4)	Yes	Yes
Northern Ontario School of Medicine	compensation, consulting, curriculum, disclosure, ghostwriting, gifts, honoraria, on-site education, sales representatives, samples, scholarships, speaking			0 (0)	No	No

**Table 3:** Number (%) of Canadian medical schools with policies in each category and strength of policy.

Category	No. of schools (%) with no policy or permissive policy (score = 0)	No. of schools (%) with moderate policy (score = 1)	No. of schools (%) withrestrictive policy (score = 2)	Mean score
**Ghostwriting**	9(53)	0 (0)	8 (47)	0.9
**Disclosure**	2 (12)	14 (82)	1 (6)	0.9
**Gifts**	8 (47)	5 (29)	4 (24)	0.8
**Scholarships**	9 (53)	2 (12)	6 (35)	0.8
**Consulting**	8 (47)	6 (35)	3 (18)	0.7
**On-site education**	9(53)	5 (29)	3 (18)	0.6
**Compensation**	10 (59)	4 (24)	3 (18)	0.6
**Honoraria**	9 (53)	6 (35)	2 (12)	0.6
**Curriculum**	12 (70)	4 (24)	1 (6)	0.4
**Speaking**	12 (70)	3 (18)	2 (12)	0.4
**Sales reps**	12 (70)	5 (29)	0 (0)	0.3
**Samples**	14 (82)	3 (18)	0 (0)	0.2

Fifteen of 17 schools had policies that identified a party responsible for enforcement of the policies (Table 2). Examples of responsible parties included “Department Head or equivalent” and “Department Chair, Dean or immediate supervisor.”

Eleven of 17 schools had policies that specified sanctions for noncompliance (Table 2). An example of such a policy from McGill University contains sanctions ranging from counselling of the individual involved all the way to termination for cause. Ten schools had policies that met requirements for both a specific party responsible for enforcement and specified sanctions for non-compliance.

## Discussion

The 17 Canadian medical schools received scores that ranged from 0 to 19 out of a possible maximum score of 24. The score of 0 was received by NOSM. This low score may reflect, in part, the fact that the school was only established in 2005. Western University received the highest score of 19. Of the 17 medical schools in Canada, over half (10) received summative scores of 5 or less out of 24, indicating that in most of the categories they had either no policy or a permissive policy. No single category managed to achieve an average score of 1 or more.

Fourteen (82%) of the schools received a rating of 0 (no policy or permissive policy) for samples. Samples have been shown to influence medical residents’ prescribing practices, with negative implications both for costs and prescribing appropriateness. Adair and Holmgren have shown that access to drug samples increases the likelihood that physicians will prescribe heavily advertised and more costly drugs as opposed to cheaper or over-the-counter drugs. [17] We also found that most medical faculties (70%) had permissive policies or no policy concerning faculty involvement in companies’ speakers’ bureaus. The United States (US) Institute of Medicine’s recent report on COI recommended banning such relationships [18] because speakers’ bureaus represent part of a company’s promotional activities and the content is often under the company’s control. [19].

Similarly, 70% of medical faculties had permissive or no policies concerning interactions with sales representatives. Sales representatives have been found to negatively influence prescribing practices, e.g., to lead to more frequent and expensive prescribing and poorer prescribing quality. [20] In a comparative study, recently graduated internists who had studied in a program that restricted contact with sales representatives were more critical of the information they provided and saw sales representatives less often than internists from a medical school without such restrictions. [21] Most schools (70%) also failed to cover conflicts of interest or drug promotion in the curriculum. This gap has important implications for students’ abilities to understand the context within which promotional activities occur and to weigh their own responses to ethical challenges that might arise. [22] Finally, nearly all schools had a party responsible for enforcing their policies (15/17) and the majority had sanctions for violations (10/17), but we do not have information on how often these sanctions are applied or how effective they are.

We found that COI policies were most stringent in the areas of disclosure, ghostwriting, gifts, (considered to be the easiest to prohibit [23]) and scholarships. These results parallel findings that AMSA obtained in its annual reviews of policies in US medical and osteopathic schools. Its 2012 analysis found that the policy areas that received the highest ratings were those that addressed scholarships, off-campus continuing medical education, purchasing, and gifts. [13] The importance of restricting gifts is emphasized in a review of COI policies at 14 American medical schools that found that exposure to a gift restriction policy during medical school was associated with reduced prescribing of two out of three newly introduced psychotropic medications. [24].

Our findings on ghostwriting are consistent with those of Chimonas and colleagues, even though their rating scale separated out no policy (score = 0) and permissive policies (score = 1). [14] They found that, although existing ghostwriting policies at American medical schools were among the most stringent of all of the policy areas, ghostwriting was also the most neglected policy area. Furthermore, other work has shown that meaningful sanctions for academic fraud are generally absent. [25] Because universities reward academic faculty for their publication records, limited enforcement can mean that faculty may find themselves complicit in ghostwriting activities, in spite of policies prohibiting them.

A similar study of Australian medical schools found that their COI policies were even weaker than those at Canadian schools. Eleven out of 15 schools received less than 50% of the maximum possible number of points and only one barely exceeded 66%. All schools either had no policies or had policies that were unlikely to have a substantial effect on behavior in the areas of on- and off-campus educational activities. Lastly, policies on consulting relationships and disclosure had mean scores below 50%. [15].

Our study, in conjunction with the ongoing AMSA survey, the analyses of the US schools by Chimonas and colleagues, and the results from the Australian schools, clearly establishes that the poor control of COI at medical schools is not confined to a single country, but is an issue that needs to be addressed at both national and international levels. One effort to engage medical students in these issues has come from a collaboration between the World Health Organization and Health Action International that has resulted in a manual to teach medical students about pharmaceutical promotion [26]. The manual is available in English, French, Russian and Spanish, and has been distributed across a wide range of countries.

This study has some limitations. Two schools did not respond to our initial request for any policies that we might have missed in our web search. Six medical schools failed to review our ratings despite repeat requests; their input could have validated, or alternatively, contradicted our findings. Furthermore, only medical schools’ COI policies were within the scope of our study, so we did not consider the policies of affiliated teaching hospitals (e.g., on samples or sales representatives). Hospitals may have had more restrictive policies, but this is unlikely based on previous research. [27].

Policy development is a dynamic process, and some Canadian medical schools have introduced new policies since September 2011, while others continue to revise their policies. It is important for medical schools to continue to develop and improve their COI policies to mitigate institution-industry relationships and to address the ways in which those relationships may affect the information that is taught to, and the attitudes of, medical students. Policies must also continue to develop, especially since the role of industry within universities continues to evolve. [23].

Practices that were once entrenched into medical culture, including the receipt of gifts, food, and drug samples, in addition to faculty consulting and speaking engagements with industry, [28] should no longer play direct or indirect roles in the education of medical students. Student-industry interactions can influence students’ education. [29] Students who have more contact with industry tend to have more favorable attitudes towards these types of interactions. [30] It has been reported that students who receive gifts from industry feel obliged to rely on industry representatives for information on medications. [31].

More stringent policies are not the only answer for helping to ensure medical education is free from faculty COI, but such policies have been shown to limit the acceptability of promotional items. [29], [32] Medical schools across Canada are encouraged to achieve the most effective and stringent policies to regulate industry relations with both faculty and students.

## Supporting Information

Appendix S1 **Grading System for Categories in Policies.**
(DOCX)
