# Supplementary material for: Too Few, Too Weak: Conflict of Interest Policies at Canadian Medical Schools
Source: PLoS One. 2013 Jul 4;8(7):e68633. doi: 10.1371/journal.pone.0068633 (PMC3701639; doi:10.1371/journal.pone.0068633)
Supplement: Appendix S1 — Grading System for Categories in Policies. (DOCX) [file pone.0068633.s001.docx]

**Appendix S1: Grading System for Categories in Policies**

**1. Gifts (including meals)**

2 = All gifts and on-site meals funded by industry are prohibited, regardless of nature or value.

1 = Less stringent limitation on industry-funded gifts (e.g., gifts prohibited above $50/year – or gifts prohibited but meals allowed)

0 = No policy, or policy that would not substantially reduce gifting (e.g., gifts are allowed but discouraged, or limited in a non-specific way to “appropriate,” or primarily for the benefit of patients).

**2. Consulting relationships (excluding scientific research and speaking)**

2 = Consulting relationships with industry must be subjected to institutional review or approval. Additionally, they must either be described in a formal contract, or payment for services must be commensurate to the task.

1 = As above, without the institutional review or approval requirement.

0 = No policy, or policy that would allow consulting relationships to occur without institutional scrutiny or that would allow relationships in which payments are not commensurate with work.

**3. Industry-funded speaking relationships/speakers’ bureaus**

2 = Speaking relationships are prevented from functioning as *de facto* gifts or marketing. An effective policy must not implicitly permit (a) long-term speaking agreements or (b) industry to have a role in determining presentation content. (Some effective policies may explicitly prohibit participation in a speakers’ bureau. Other effective policies contain elements such as limits on compensation and reimbursement and a requirement to ensure the scientific integrity of information presented.)

1 = Industry-funded speaking relationships are regulated, but with less stringent limits on longevity, content or compensation.

0 = No policy, or policy that does not define the limits on longevity, content or compensation.

**4. Honoraria**

2 = No acceptance of honoraria; compensation must be at fair market value and publicly disclosed

1 = Limits on accepting/disclosing honoraria.

0 = No policy, or no limits on acceptance.

**5. Ghostwriting**

2 = Ghostwriting is not permitted.

1 = Few or no restrictions; management is left to individual discretion.

0 = No policy.

**6. Disclosure**

2 = Personnel are required to disclose past and present financial ties with industry (e.g., consulting and speaking agreements, research grants) on a publicly-available website and/or disclose such relationships to patients when such a relationship might represent an apparent conflict of interest.

1 = Universally-required, internal disclosure to the medical school or hospital administration. (Policies requiring disclosure only when presenting or publishing do not meet this criterion.)

0 = No policy.

**7. Industry Sales Representatives**

2 = Pharmaceutical and device representatives are not allowed to meet with faculty regardless of location, or are not permitted to market their products anywhere inside the medical center and associated clinics and offices. (Exceptions may be made for non-marketing purposes, such as training on devices or equipment.)

1 = Pharmaceutical representatives are permitted to meet with faculty, but with significant limitations (e.g., only in non-patient care areas or only by appointment). Exceptions as above.

0 = No policy, or policy that does not substantially limit access.

**8. On-site Education Activities**

2 = Industry is not permitted to provide direct financial support for educational activities, including Continuing Medical Education (CME), directly or through a subsidiary agency.  (However, companies may contribute unrestricted funds to a central fund or oversight body at the academic medical center, which, in turn, would pool and disburse funds for programs that are independent of any industry input or control.)

1 = Less stringent limitations to ensure independence of educational content (e.g., standards to establish freedom from industry influence of content, such as review and approval of presentations; language that prevents industry from selecting the speaker; or language such as: industry funding may be allocated for a particular topic, but must be provided directly to the department, not to individuals).

0 = No policy, or a policy that would not substantially limit industry influence over educational activities (e.g., industry funding must be disclosed).

**9. Compensation for Travel or Attendance at Off-site Lectures & Meetings**

2 = Personnel may not accept payment, gifts or financial support from industry to attend lectures and meetings. (An exception may be made for modest meals, if part of a larger program.) Travel support may only be accepted if it is subject to institutional approval or industry is prevented from selecting (“earmarking”) the recipients. Note: speaking and consulting relationships are evaluated separately in domain 1.

1 = Less stringent limitations.

0 = No policy, or a policy that would not substantially limit participation in industry-funded events and meetings.

**10. Industry Support for Scholarships & Funds for Trainees**

2 = The policy must either prevent industry from earmarking or awarding funds to support the training of particular individuals (recipients must be chosen by the school or department), or the policy must mandate institutional review of the giving of funds. (This does not preclude grants that fund a specific research project.)

1 = Less stringent limitations.

0 = No policy, or a policy that would not substantially regulate industry funding of scholarships and funds for trainees.

**11. Medical school curriculum (or other documentation of educational objectives/course content)**

2 = Students are trained to understand institutional conflict-of-interest policies and recognize how industry promotion can influence clinical judgment.

1 = Curriculum addresses conflict of interest in a more limited way (e.g., training on policies only).

0 = No policy (not addressed in curriculum or elsewhere).

**12. Samples**

2 = Industry samples are prohibited, except under certain narrow circumstances approved by the institution that protects the interests of patients and prevent the use of samples as a marketing tool (e.g., policies that allow samples under limited circumstances with the approval of the Pharmacy and Therapeutics (P&T) Committee or policies that incorporate samples into a larger program designed to ensure the availability of brand-name and generic medications to underinsured patients; if the circumstances of the specific program are not defined, the policy should define the approvals process). Where there is a specific program in place, the policy must prevent samples from being given directly to physicians by pharmaceutical sales representatives. Samples must not be for the personal use by physicians.

1 = Samples or vouchers for medications may be provided, but with significant limitations (e.g., samples may not be given directly to physicians, samples must be dispensed or controlled by pharmacy department).

0 = No policy, or a policy that does not substantially limit the use of samples (e.g., samples limited for formulary items or samples not for personal use.

**13. Enforcement**

A. Is it clear that there is a party responsible for general oversight to ensure compliance?  (Y/N)

B. Is it clear there are sanctions for noncompliance?  (Y/N)
